# Investigating the impact of a large river and its surrounding contextual conditions on pedestrians’ summer thermal perceptions in a Cfa-climate city

**DOI:** 10.1038/s41598-024-64729-7

**Published:** 2024-06-15

**Authors:** Ting Liu, Siqi Wang, Jian Zhang, Lintai He, Bin Cheng, Huiyun Peng, Fanchun Liu, Bo Tan, Xiaowei Shang, Fan Liu

**Affiliations:** 1 School of Civil Engineering and Architecture, Southwest University of Science and Technology, Mianyang, China; 2 Mianyang Municipal Bureau of Natural Resources and Planning, Mianyang, China; 3 Sichuan Changhong Development, Mianyang, China

**Keywords:** Outdoor thermal comfort, Neutral temperature, Large water surfaces, Distance from the water, Microenvironment, Biophysics, Climate sciences, Environmental sciences

## Abstract

Thermal comfort studies are usually employed to find subjective thermal responses [indicated by neutral temperature (NT), i.e. the temperature with no thermal stress] of residents from a region towards thermal environments. According to the recently published works in the literature, NTs are affected by many factors, such as geographical location and microenvironments. To elucidate the origins of these effects, the impact of microenvironment elements around a water surface on pedestrians’ thermal perceptions was systematically investigated in this work. The Fujiang River (FJR) in Mianyang City was taken as the sample site. The municipal meteorology station is located next to the site by around 2.5 km. By performing meteorology measurements combining questionnaires, it was found that the riverside NT (indicated by physiologically equivalent temperature, PET) of Mianyang in the summer of 2023 was 21.4 °C. The relationship between the distance from the water (DFW) and NT was quadratic linear. The same phenomenon took place by using either PET or Universal Thermal Climate Index (UTCI) indexes. Meanwhile, the meteorological contexts also affected NTs, including relative humidity (RH) and air velocity (V_a_). Regarding RH, the NPET increased from 15.2 °C (RH = 50%) to 26.9 °C (RH = 90%). In contrast, the NPET dropped from 23.0 to − 50.6 °C when the V_a_ increased from 0.2 to 2.5 m/s, respectively. From our analysis, it was demonstrated that human thermal responses are significantly affected by both the microenvironmental and meteorological backgrounds around the water surface. Our work provides valuable insights for the proper use of water surfaces in urban design for adjusting thermal comfort.

## Introduction

The global warming and urban heat island (UHI) problems undoubtedly affect people's life quality and city livability^1^. In recent years, extremely high temperatures have frequently emerged in many areas around the world. This effect has caused a series of social-related issues including poor health^2^, thermal discomforts^3^, and psychological stress^4^. Consequently, residents need to rely on mechanical equipment for thermal adjustment^5^, which causes a large amount of energy consumption^6^, resulting in energy crisis^7^ and economic pressure^8^. Therefore, improving thermal comforts in energy-free ways has been an integral task of the sustainable development process^9^. Various strategies relating to this process have been deployed in recent years.

Several approaches have been explored to reduce the dissipated heat stress. The studies related to this effect in outdoor spaces are defined as outdoor thermal comfort (OTC^10^). More specifically, the goal of OTC studies involving subjects is to improve the comfort level of humans. Subjective responses towards certain thermal conditions are exported, which is supported by professional techniques. This approach involves subjective factors and objective (thermal) environments. Subjective perceptions are usually numerically modelled. This can be evaluated by the thermal sensation vote (TSV) factor, which typically classifies thermal levels into 7 scales ranging from -3 (cold) to 3 (hot)^11^. Human thermal responses on TSV are usually positively correlated with the heat levels (temperature). For instance, pedestrians’ responses increased from 0 to 3 as the temperature increased from 23.3 to 39.3 °C^12^. The thermal perception is affected by diverse meteorology parameters (air temperature (Ta); air velocity (V_a_); relative humidity (RH), etc.). To take into account this effect, various complex indices containing a variety of meteorological parameters have been developed^13^. Particularly, physiologically equivalent temperature (PET^14^), standard effective temperature* (SET*^15^), and universal thermal climate index (UTCI^16^) have been proposed and frequently used for thermal environments. The PET index was used by Liu et al.^12^ (abovementioned).

The subjective thermal responses are the key findings of the OTC-related studies. They are usually indicated by neutral temperature (NT, the temperature point with no thermal stress). TSV is significantly linearly associated with thermal indices, which supports the calculation of NT. The NT has been extensively explored in recent decades and has been found to vary regionally. The NPETs values (the NT indicated by PET) in Chongqing^17^, Xiamen^18^, and Sydney^19^ were 23.6 °C, 24.7 °C, and 26.2 °C, respectively. The recorded heat perceptions varied also during the various seasons. Liu et al.^12^ found that NPET in summer was higher than that in winter.

The NTs have been also thoroughly explored in the last decades. In recent years, OTC-related studies have focused on extra factors that would affect the subjective perceptions, such as participants’ origins^20^, age^21^, physical activity^22^, etc. The impact of these factors was revealed by the variation of NT. People’s thermal responses also varied for latitude in the macro scale^23^. All these factors significantly affect the thermal sensations, in both micro or macro scales^24^. The variation of NTs among the various factors was also confirmed. The impact of a variety of microenvironmental factors (water surfaces^4^, site openness^25^, vegetation^26^, and so on) has been also corroborated. It could be deduced that humans would thermally adapt to them. This assumption has been verified by Xiong et al.^27^ in the water surfaces. Nevertheless, further exploration is required to elaborate on the origins of the above-mentioned effect. Along these lines, in this work, the impact of a large water surface (river) on pedestrians’ thermal responses (indicated by NTs) was systematically studied. This study would be helpful to determine the influence of urban water surfaces on the thermal comfort of people on the riverbank in the Cfa climate areas, which is practical impactive for improving the outdoor thermal comfort by urban design at waterfront spaces. The whole work was conducted by the following steps:

- A large water surface (river) was found that might be microclimatic influential in the city as the sample.
- A variety of points around the waterbody were selected that would be meteorologically affected by the different degrees of the field measurement.
- The data at each site by field surveys were collected (meteorological parameters by sensors, subjective information by questionnaire, and physical environments by WinSCANOPY^28^).
- The data of different aspects were associated with each other to find their statistical associations.

## Methodology

### The sample city

**Figure 1:**
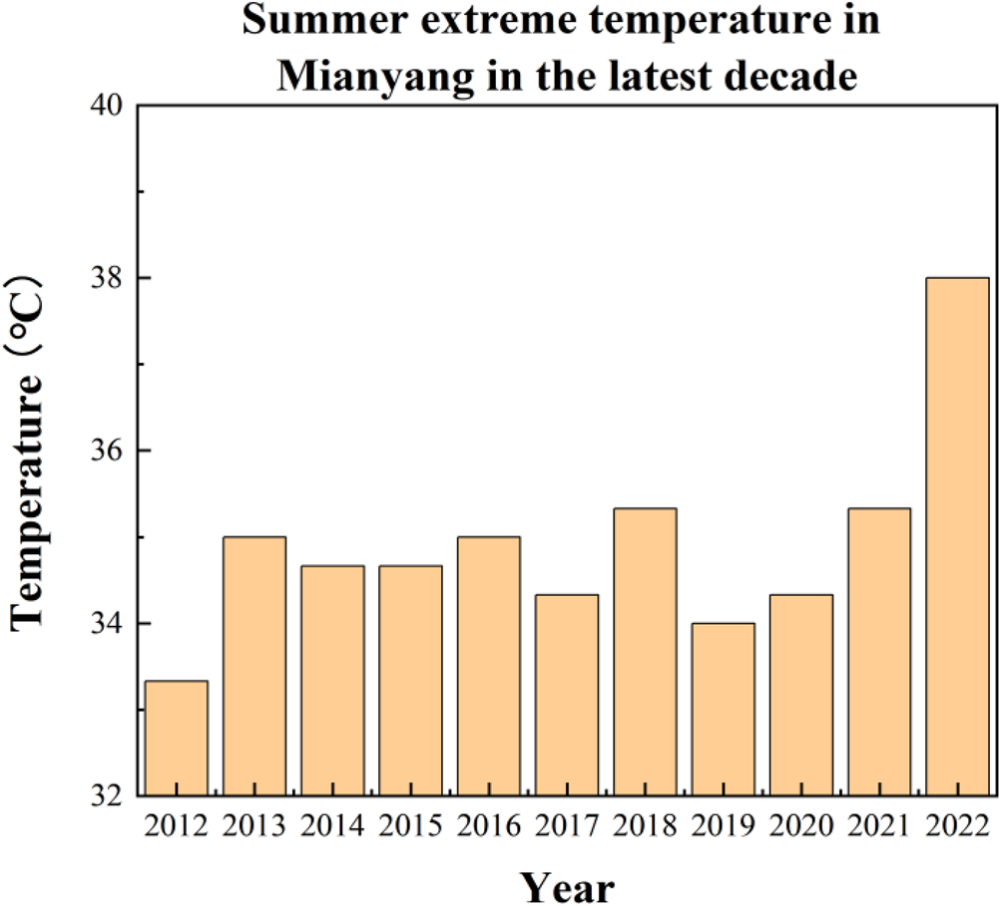
Histogram of extreme temperature values recorded in Mianyang in the latest decade in summer.

The Cfa refers to the climate type with the subtropical humid meteorology^29^. It is cold in winter and hot in summer with high RH in warm seasons. This climate is very common in southern China^30^. Mianyang is located in the southwest of China is a typical Cfa city. With the continuous urban environment degradation, such as pollution^31^ and natural land shrinkage^32^, extreme weather conditions have frequently appeared in recent years^33^. The summer of Mianyang city spanned from June to Early September every year; The annual hottest month has a maximum temperature above 35.0 °C (Fig. 1^34^). Nevertheless, recently, the summers are gradually getting warmed. The year 2022 has experienced the hottest summer of Mianyang in history (Fig. 1). Therefore, avoiding the impact of extreme weather is currently emerging as an urgent task.

**Figure 2:**
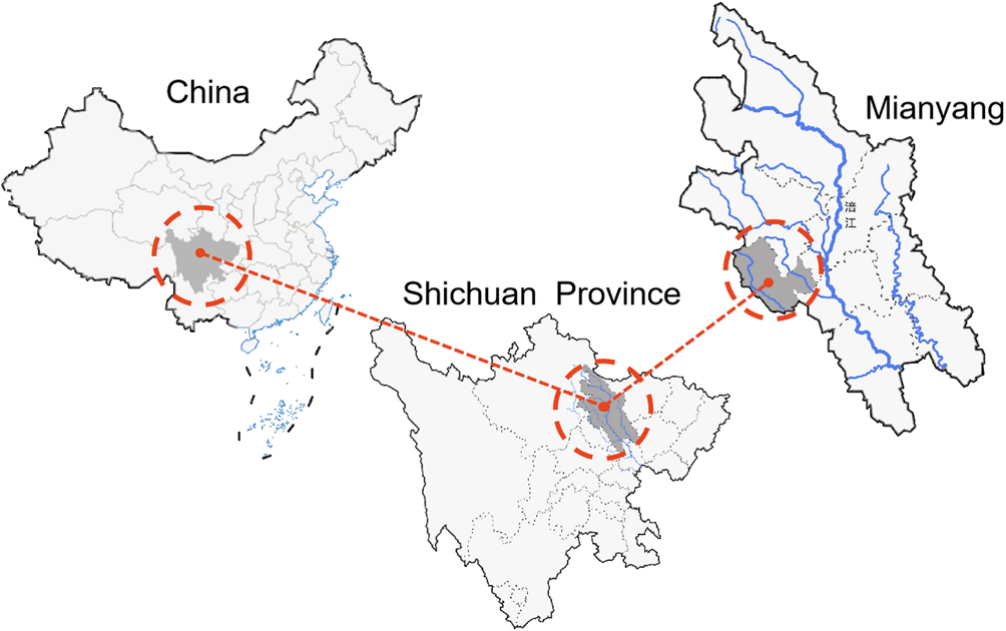
Locations of the city and the studying areas^35^ (processed by Adobe Photoshop^40^).

**Figure 3:**
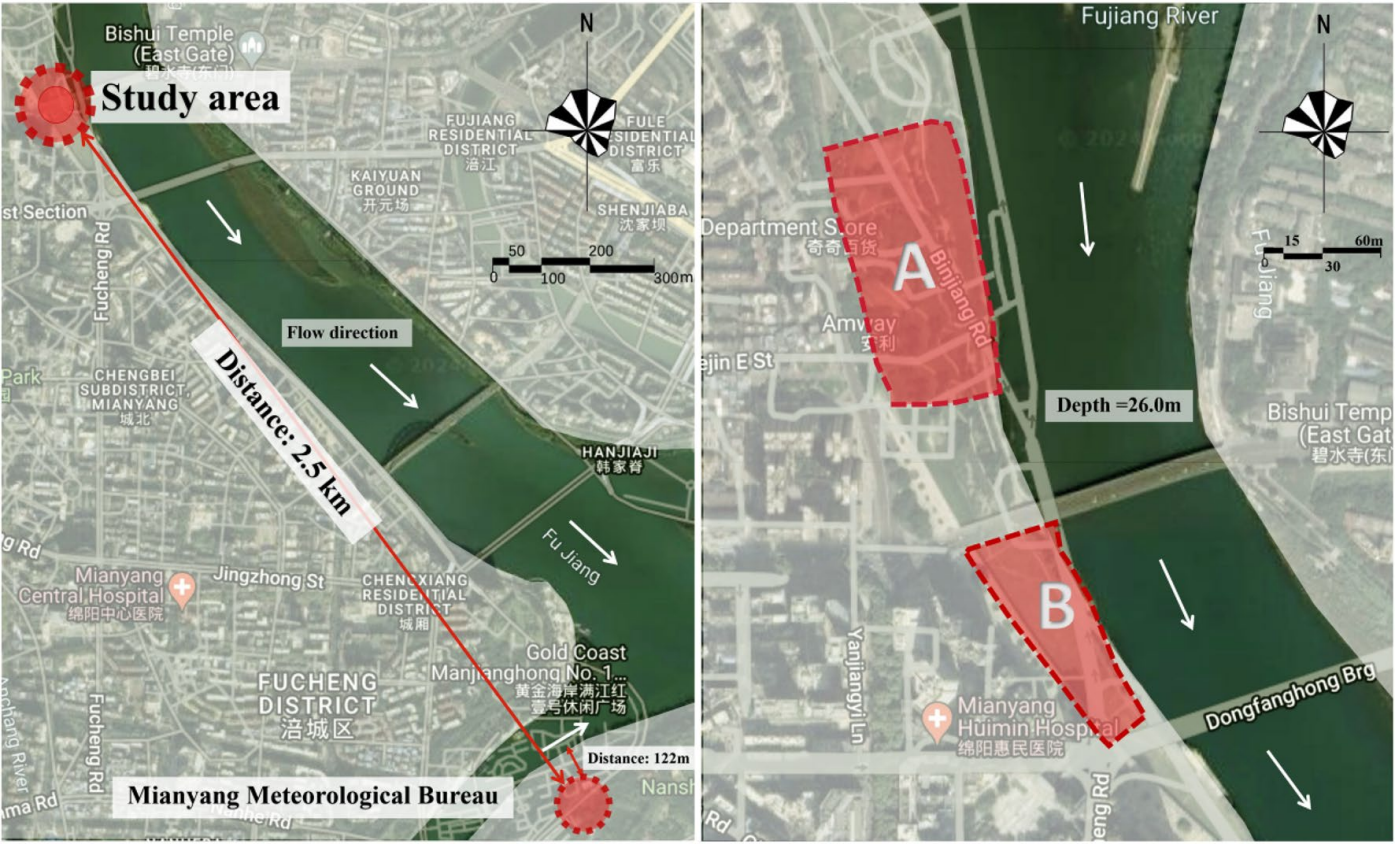
Locations of the 2 sites^35^ (processed by Adobe Photoshop^40^).

Mianyang is located in the north of Sichuan, China (Fig. 2), and is geographically surrounded by mountains. A big river (the Fujiang River, FJR) goes through the city. The width of the river section in the study area is 228.0 m, the maximum depth is above 26.0 m, and it flows from northwest to southeast (Fig. 3)^35^. The cooling effect of the water surfaces has been widely confirmed to be effective^36^. The cooling intensities are strongly dependent on the local physical properties^37^ and could be also affected by the attributes of FJR. FJR supplements the local water demands while adjusting the city climate. In the present work, the sites around the riverbank were selected as samples for field measurement. According to the literature, it has been confirmed that waterbodies and site geometry affect the thermal comfort levels. As a result, the sites located closer to the water surfaces^38^ and/or with lower openness^39^ are cooler.

Therefore, points with different distances from the water surface (DFW) on the bank were selected for performing the measurements. There were a total of 16 points chosen, which were divided into two groups (Groups A & B). The test site was around 2.5 km from Mianyang Meteorological Station (Fig. 3). Group A refers to sites canopied by trees, whereas the B were mostly open. All points in each group ranged on a line being vertical to the river. They were defined as Points 1 (within 5 m of the river) to 8. The neighboring points were 10.0 m to each other.

**Figure 4:**
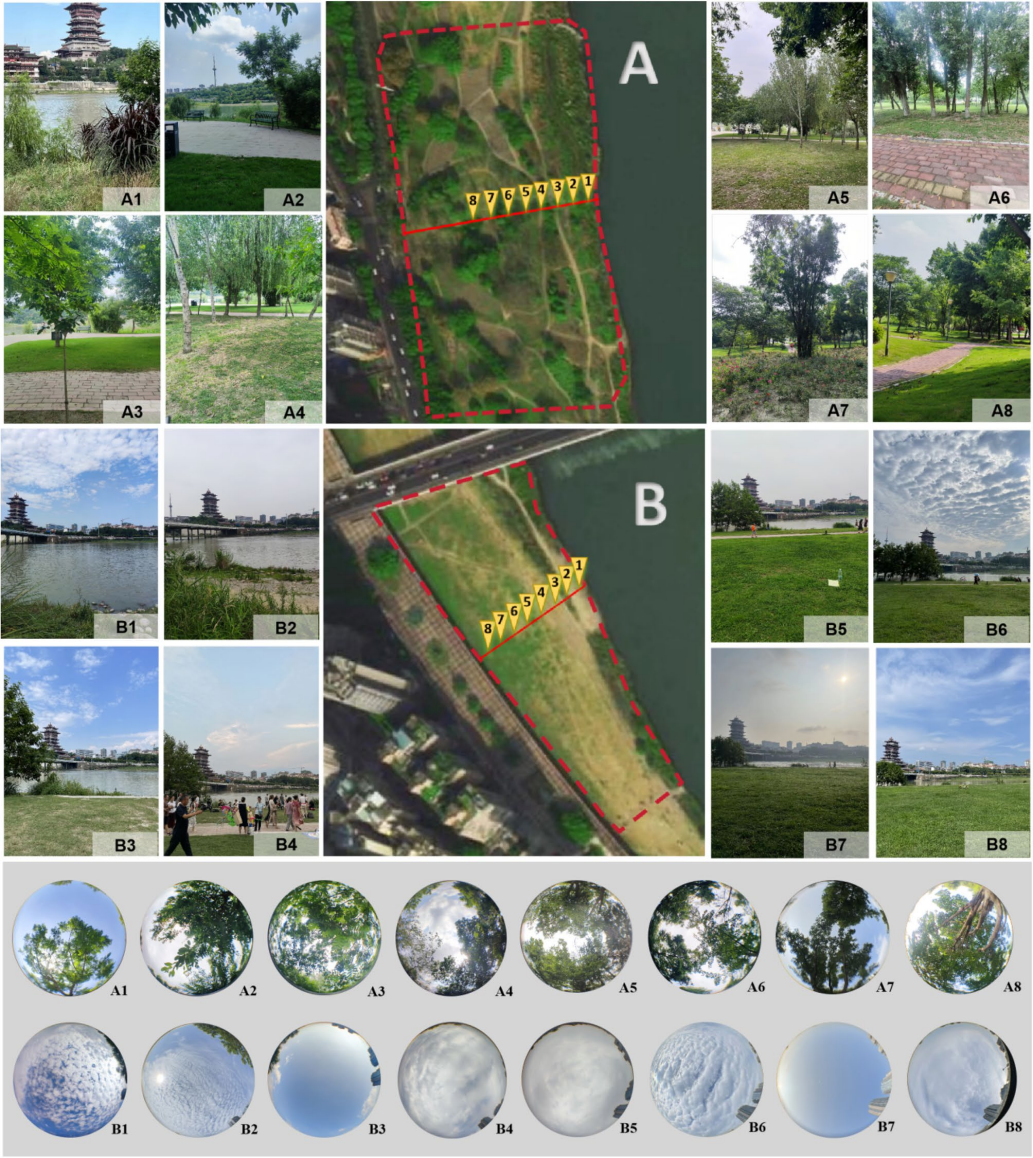
Locations, surrounding environments, and sky view factor images of all points (processed by Adobe Photoshop^40^).

The representative Groups A (canopied) and B (open) along the FJR were selected as samples. According to the degree of vegetation cover, the sites can be classified as open (without canopy cover) or closed (densely covered by trees)^41^. Their coverage was indicated by the sky view factor (SVF^42^). The SVF is a parameter developed by WinSCANOPY^28^ supporting the forestry and agriculture studies. It defines the site openness by the visibility of the sky in a hemisphere, ranging from 0 (fully covered) to 1 (totally open). The values of this factor could be acquired from the software in WinSCANOPY. The points in Group A have lower SVF than B; the lands on two lines were covered lawns. They were defined as A1 to A8 and B1 to B8. Figure 4 presents the images of the physical characteristics of all points (locations, surrounding environments, & SVF).

### Field measurement

#### Meteorology measurement

The measurements were carried out between 16 and 23 July 2023, which was the annual hottest time. All measurement days were cloudless. There were two sets of Testo 480 supporting the meteorology data collection including T_a_, V_a_, RH, and globe temperature (T_g_). They were implemented by different sensors and their properties are listed in Table 1. Every point was measured by one full daytime (8:00 a.m.–8:00 p.m.). The two points were fully measured each day. The measuring information of each point is presented in Table 2. Table 3 lists the analyses of standard deviation for the meteorology data.

**Table 1:** Properties of all sensors of Testo 480^43^.

Sensors	Measuring range	Accuracy	Resolution
Air velocity	0–20 m/s	± (0.03 + 0.5%)	0.01 m/s
Relative humidity	0–100%	± (1.8%RH + 0.7%)	0.1%
Globe temperature	0–120 °C	± (0.3 + 0.1%)	0.1 °C
Air temperature	0–50 °C	± 0.5 °C	0.1 °C

**Table 2:** The measurement date and time.

Date (2023)	Point	Maximum (℃)	Minimum (℃)	Station temperature (℃)	SVF
July 16	A1	41.2	26.1	30.1	0.4
July 16	B1	47.7	24.6	30.1	0.5
July 17	A2	43.3	25.4	30.5	0.3
July 17	B2	46.7	28.5	30.5	0.5
July 18	A3	35.0	26.2	29.8	< 0.1
July 18	B3	47.0	25.2	29.8	0.5
July 19	A4	35.1	26.0	29.4	< 0.1
July 19	B4	39.9	26.6	29.4	0.6
July 20	A5	32.3	25.2	28.2	0.1
July 20	B5	44.3	25.7	28.2	0.6
July 21	A6	36.4	24.5	30.2	0.3
July 21	B6	41.8	25.2	30.2	0.4
July 22	A7	46.7	28.3	31.5	0.4
July 22	B7	39.6	27.2	31.5	0.5
July 23	A8	36.5	28.1	31.5	0.1
July 23	B8	43.4	28.5	31.5	0.6

**Table 3:** The standard deviation of meteorology for the two groups.

	Group A	Group B
Mean temperature (℃)	31.7	34.0
Temperature standard deviation	1.7	1.3
Mean relative humidity (%)	58.6	52.0
Relative humidity standard deviation	2.1	2.1
Air velocity (m/s)	0.4	0.5
Air velocity standard deviation	0.1	0.2

#### Questionnaire

Subjective information of all interviewees was obtained by questionnaire, which was carried out simultaneously with meteorology measurement. The questionnaire was divided into two parts according to the research content (Table 4). The first part was about subjective perceptions. TSV, spanning as − 3, cold; to + 3 hot; and + 4 very hot)^44^. The − 4 (very cold) was ignored as nobody would vote that in such hot weather. The setting followed ISO^44^ and ASHRAE^45^. Volunteers were suggested to vote with a resolution of 0.1 in TSV^27^. The second part was about the personal information that would affect thermal perceptions including clothing, gender, height, etc. It took about 5 min for the subjects to understand and to answer all questions. For a large number of questionnaire sheets collected, some volunteers who frequently visited the riverbank were repeatedly interviewed. A total of 1669 questionnaire sheets were obtained from the 16 points, and 1617 of them were valid supporting the analyses, with a validation rate of 96.9% (a few incomplete sheets were excluded, Table 5). All participants completed the questionnaire voluntarily and anonymously. There was no illegal harm to the participating subjects. It was also confirmed that informed consent has been obtained from all subjects and/or their legal guardians. The whole questionnaire was ethically approved by Southwest University of Science and Technology. Meanwhile, all methods via in this study were performed in accordance with relevant guidelines and regulations of this journal. The original data generated or analysed during this study are included in this published article as the supplementary (the excel file named as ‘The original data 1214’) file.

**Table 4:**
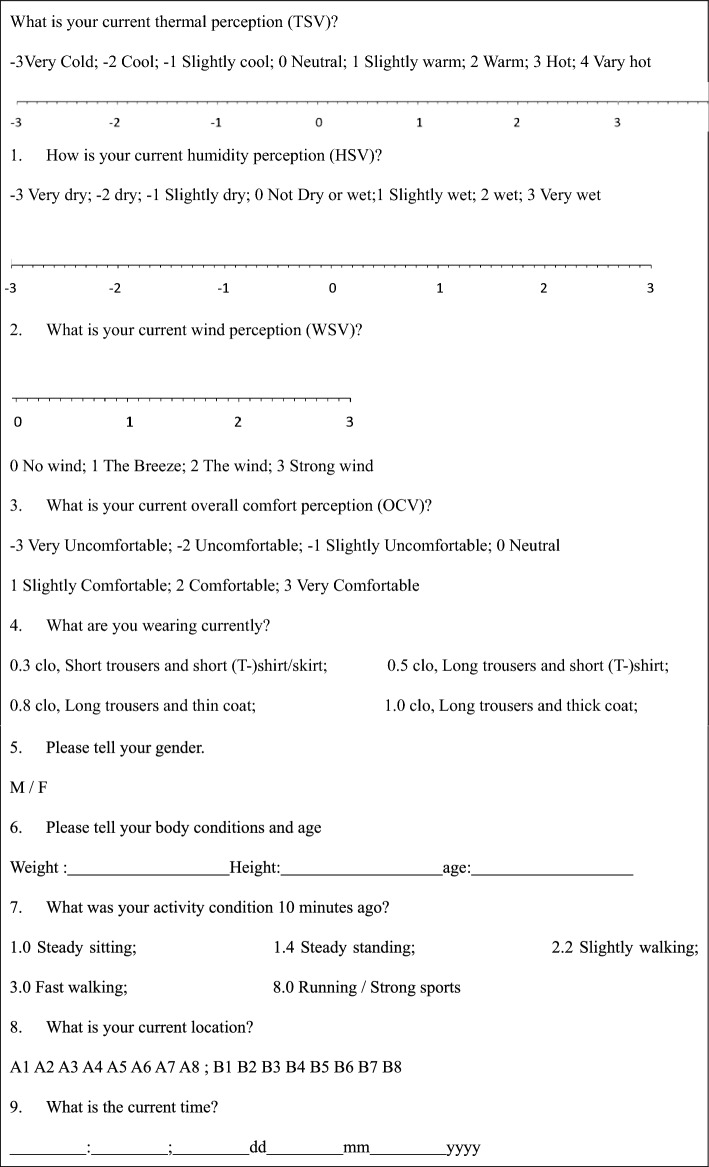
Questionnaire survey.

**Table 5:** Number of questionnaires issued and collected.

Point	Number of volunteers	Number of questionnaires collected	Point	Number of volunteers	Number of questionnaires collected
A1	103	102	B1	104	101
A2	105	102	B2	105	102
A3	103	101	B3	104	102
A4	102	102	B4	103	102
A5	105	102	B5	102	100
A6	102	102	B6	102	102
A7	103	91	B7	110	102
A8	108	102	B8	108	102

### Thermal comfort indices

The thermal comfort is affected by various parameters. To address this issue, complex indices have been proposed in the literature. They contained both meteorological parameters and volunteers’ individual factors (i.e., clothing isolation).

Normally, PET^14^ and UTCI^46^ have been frequently used. PET is based on the Munich Personal Energy Balance Model (MEMI), which simulates the thermal conditions of the human body in a physiologically relevant way. It can be defined as the T_a_ at which the human body's thermal budget in a typical indoor environment (without wind and solar radiation) is in balance with the skin temperature under complex outdoor conditions^14^ (MRT = T_a_, work metabolism = 80 W, clo = 0.9, etc.). UTCI can be defined in another s (V_a_ = 0.5 m/s, work metabolism = 135 W, RH = 50% etc.)^46^.

A large number of works in the literature have demonstrated that the perceived comfort values of PET and UTCI are very similar^16^. The common equation for MRT can be expressed as follows^47^.

$${\text{MRT}}={\left[{({\text{T}}_{\text{g}}+273.15)}^{4}+\frac{\left(1.10\times {10}^{8}{\text{v}}^{0.6}\right)\left({\text{T}}_{\text{g}}-{\text{T}}_{\text{a}}\right)}{\upvarepsilon {\text{D}}^{0.4}}\right]}^{1/4}-273.15$$

**Figure 5:**
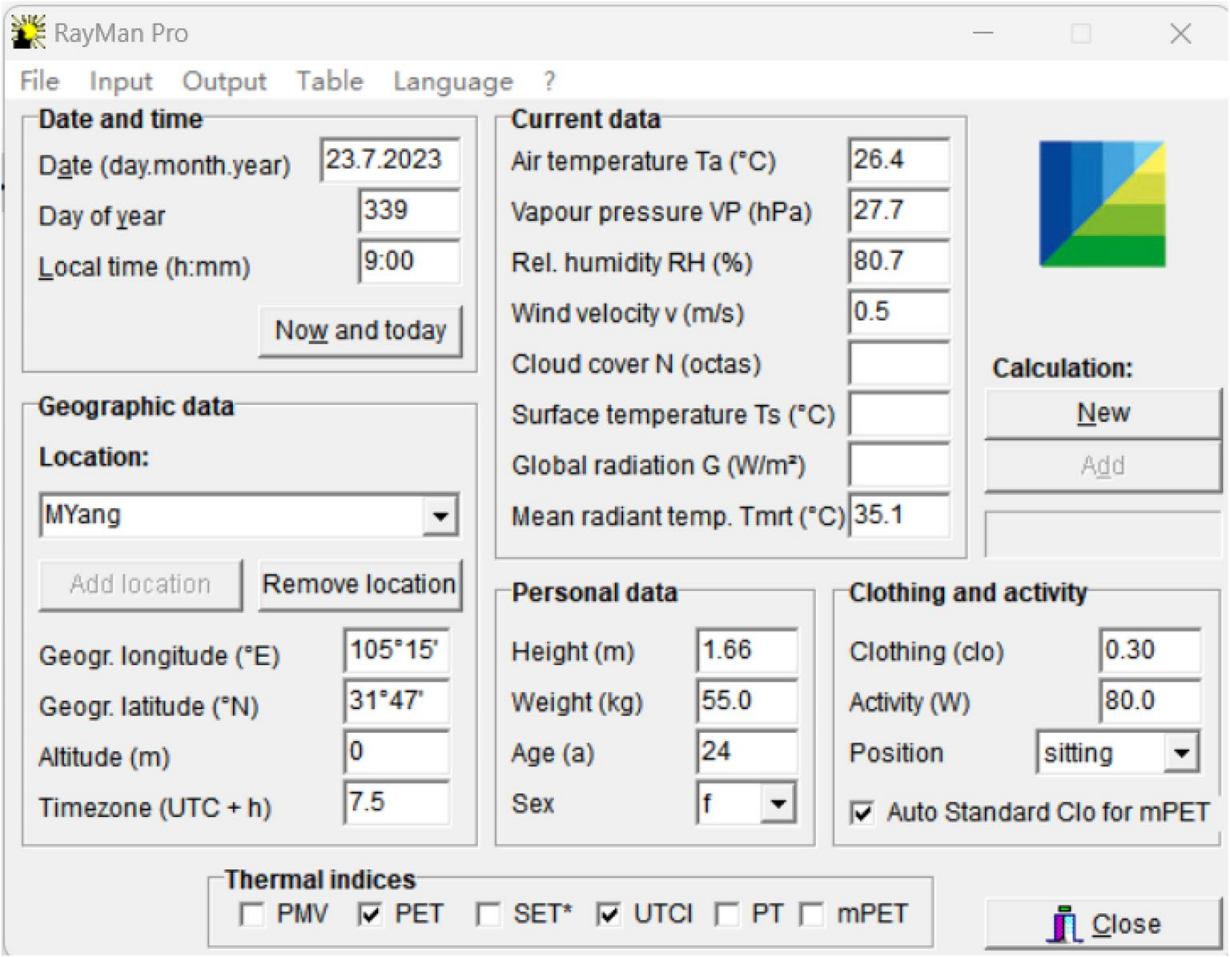
RayMan Model for the calculations of PET and UTCI.

The signs in the formula refer to MRT, T_g_, T_a_, V_a_ (v); whereas D and ε are the diameter of the globe (D = 0 0.15 m) and its absorption rate (0.95), respectively. The values of the indices have been calculated by RayMan Model^14^ (Fig. 5) and developed by Matzarakis et al.^48^. Either of the three indices has been used in OTC studies. UTCI was developed in 2009 by international co-operation between leading experts in the areas of human thermophysiology, physiological modelling, meteorology, and climatology^49^. By comparing the neutral temperature and neutral range of the PET and UTCI indices, significant differences were found between the neutral temperature and the neutral range in summer and winter^50^. The increase of MRT by 10.0 °C contributed to a TSV ascent of 0.9^51^.

### Data analyses

Multiple linear regression (MLR) is usually utilized for data analyses in OTC studies^52^. It is an analytical method that creates a linear relationship between a dependent variable and one or several independent variables. The MLR was used in this work to explore the linear correlation between thermal perception and thermal environment under the action of different microenvironmental factors. The impact of riverside microenvironmental factors on people’s thermal perceptions was explored here, which is affected by the NT variation. NTs of all points needed to be calculated by MLR. The TSV and PET were available as dependent and independent variables.

### Ethical approval

This study was ethically approved by Southwest University of Science and Technology (23zx7107).

## Results

### Data description

**Figure 6:**
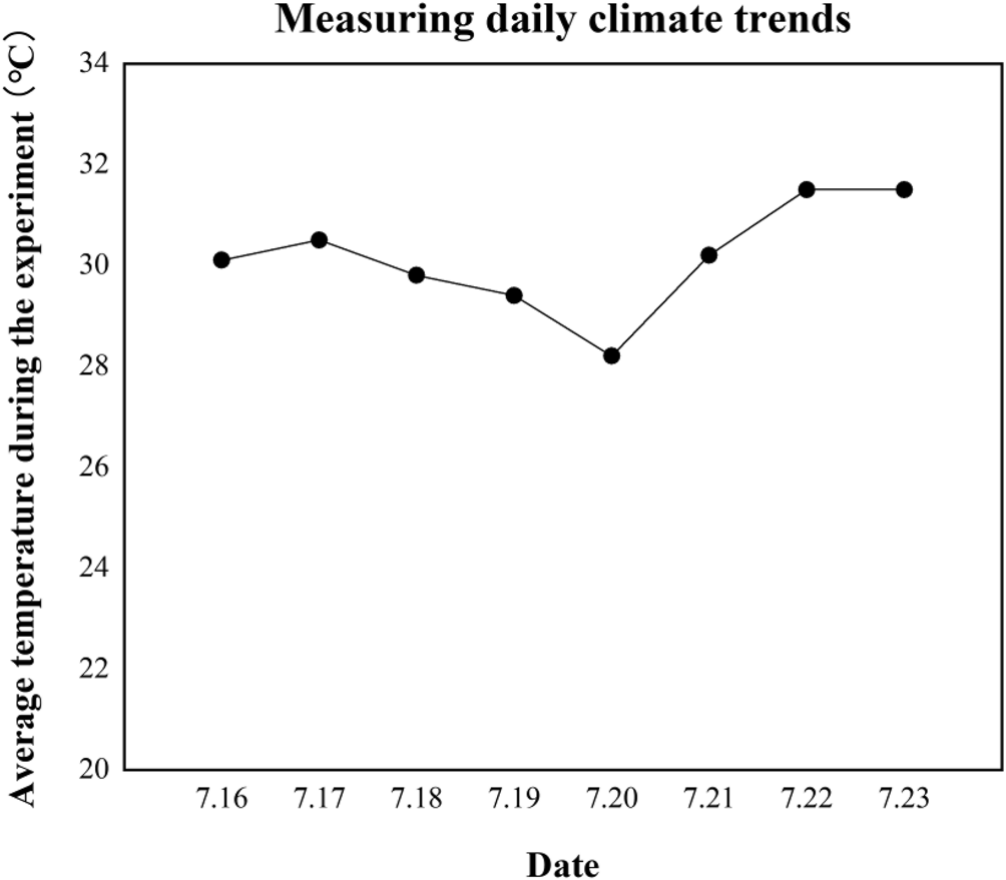
Weather changes of all measurement days by mean T_a_ from the weather station.

**Figure 7:**
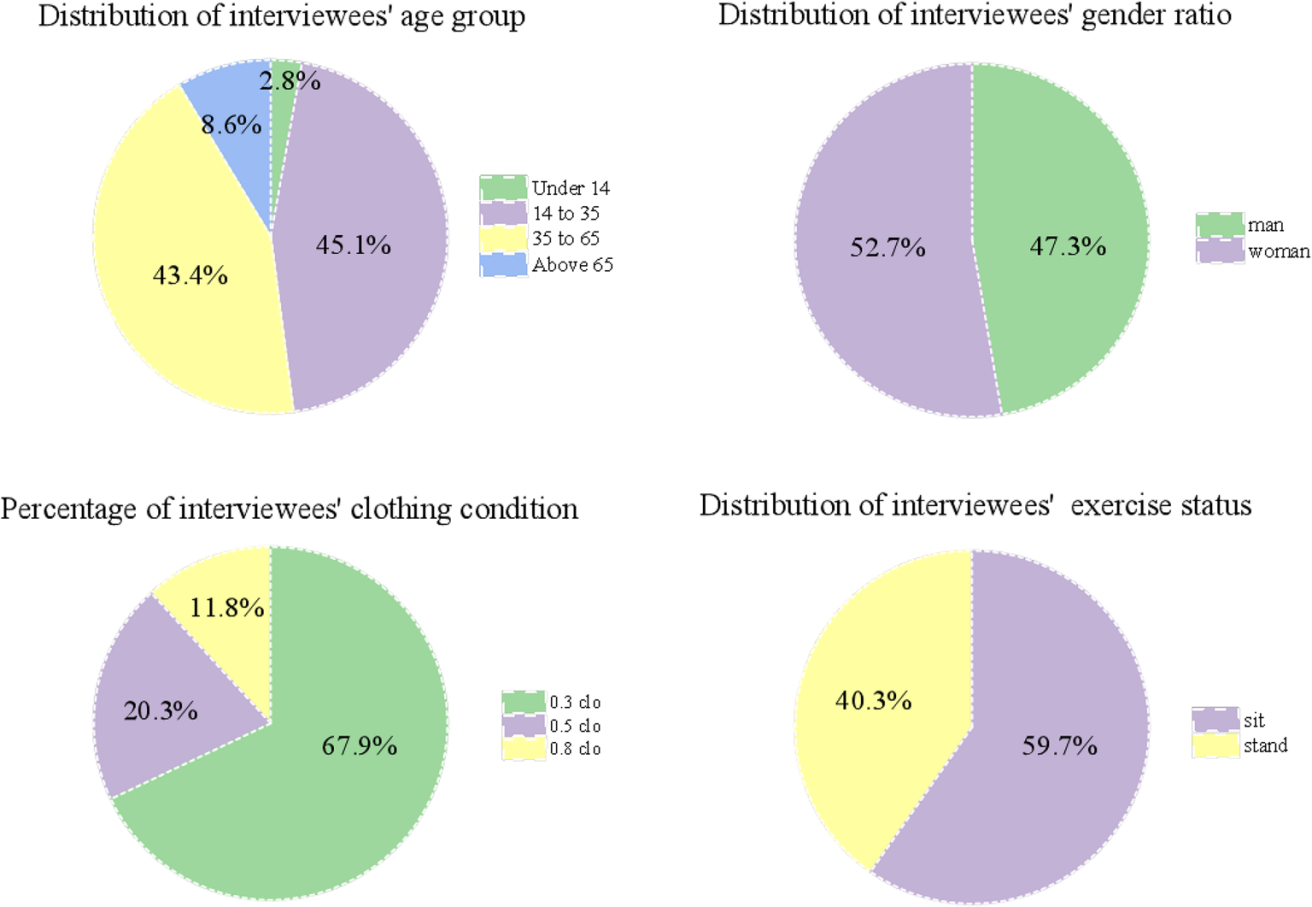
Personal information about volunteers.

Figure 6 describes the daily weather of all measurement days (8:00–20:00). As can be seen, they were totally very hot (above 25.0 °C on average). Generally, it got cool from 16 (30.0 °C) to 20 (27.0 °C) in July, yet getting warm afterwards, peaking at 31.0 °C (23 July.). Figure 7 shows the personal information of the volunteers when they received the questionnaire.

**Figure 8:**
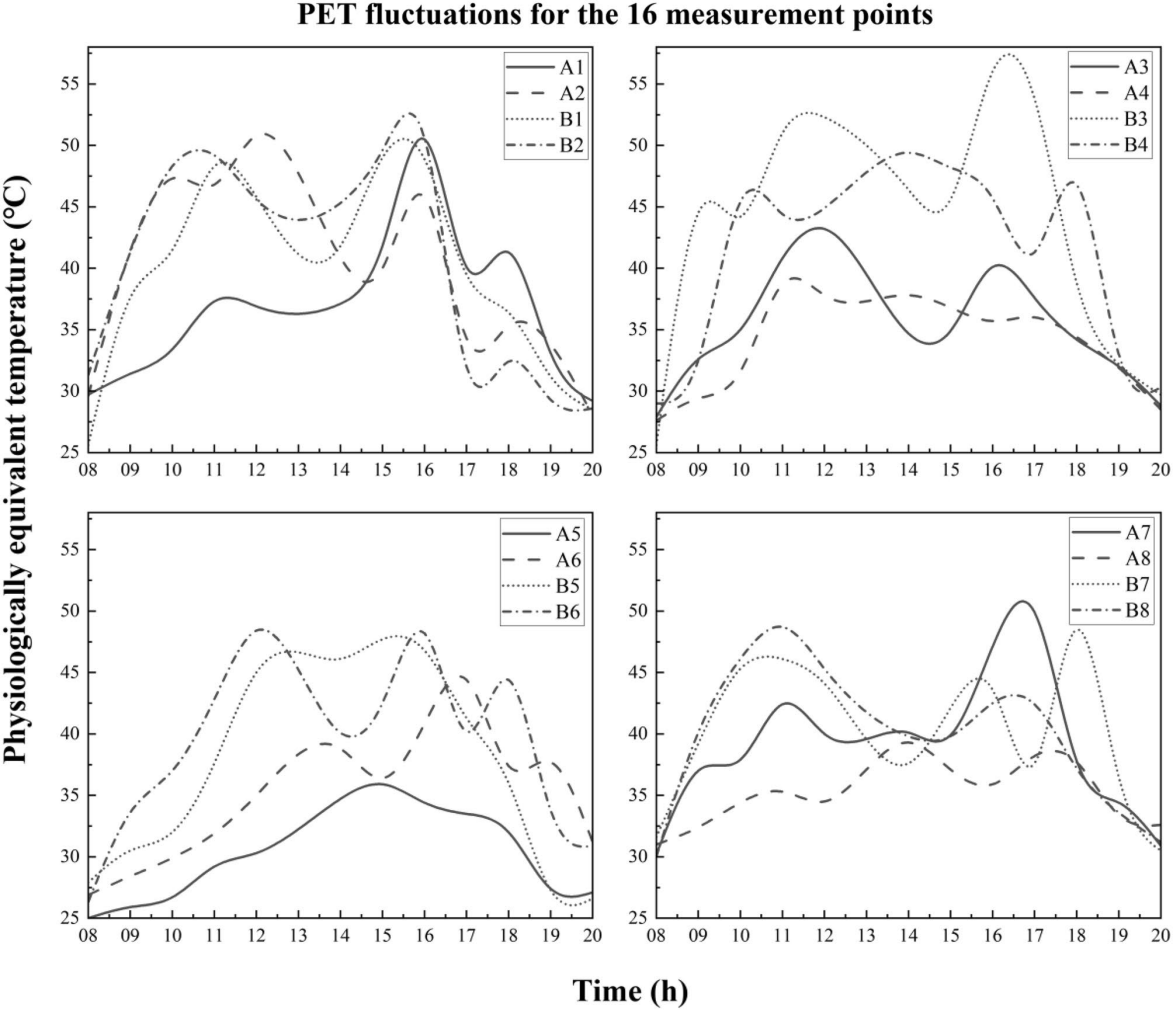
PET fluctuations for all points during the measurement periods.

Figure 8 illustrates the daily PET fluctuation of all 16 points. Each image contains 4 points measured within 2 days. Generally, Points A had lower values than the performances of tree canopies. A1 was cooler than B1 by nearly 10.0 °C at 11:00. As the daily weather changed, all PET values were irregularly changed. Most of them peaked around 17:00 (e.g., 50.0 °C for B2 & 57.0 °C for B3). Nevertheless, the points measured on the same day expressed similar trends. For instance, B6 increased from 25.0 °C (8:00) to 46.0 °C (12:00), and then reduced till 14:00 (40.0 °C). A6 expressed a similar trend despite the different extreme values. Three characteristics for all PET can be derived: (1) the existence of higher values at open points; (2) two points measured on the same day exhibited similar waves; (3) various changing regulars were recorded at all measurement days.

**Figure 9:**
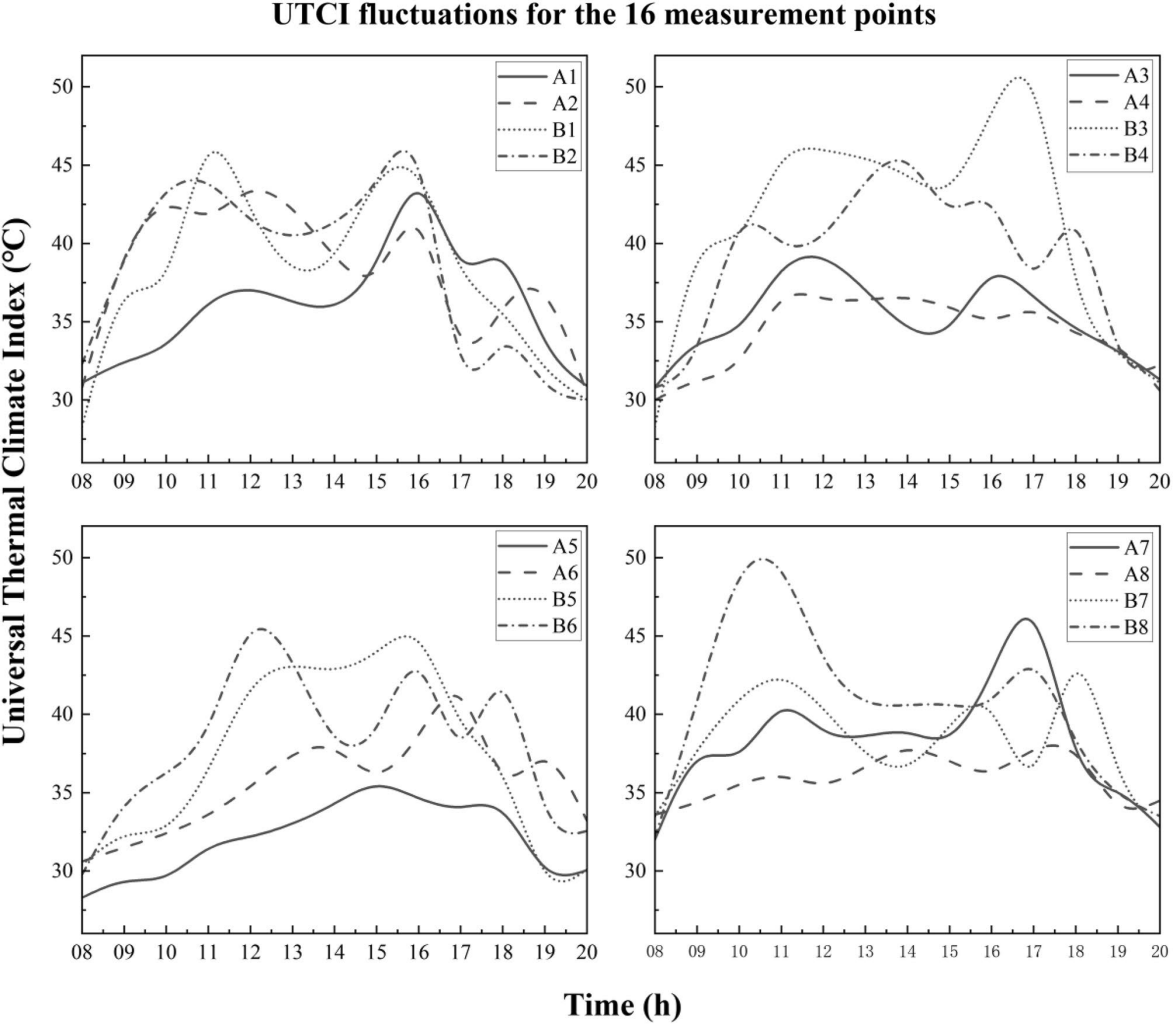
UTCI fluctuations for all points during the measurement periods.

Waving regulars of UTCI are expressed in Fig. 9. Their trends were generally similar to PET. For example, A1 waved similarly with B1, since measured on the same day (increasing from 8:00 to 12:00, bottoming at 14:00, peaking again at nearly 16:00). Open sites were warmer than canopied ones as well, such as B3 being hotter than A3 by 15.0 °C at 17:00. The values of UTCI at the points of all days fluctuated differently.

**Figure 10:**
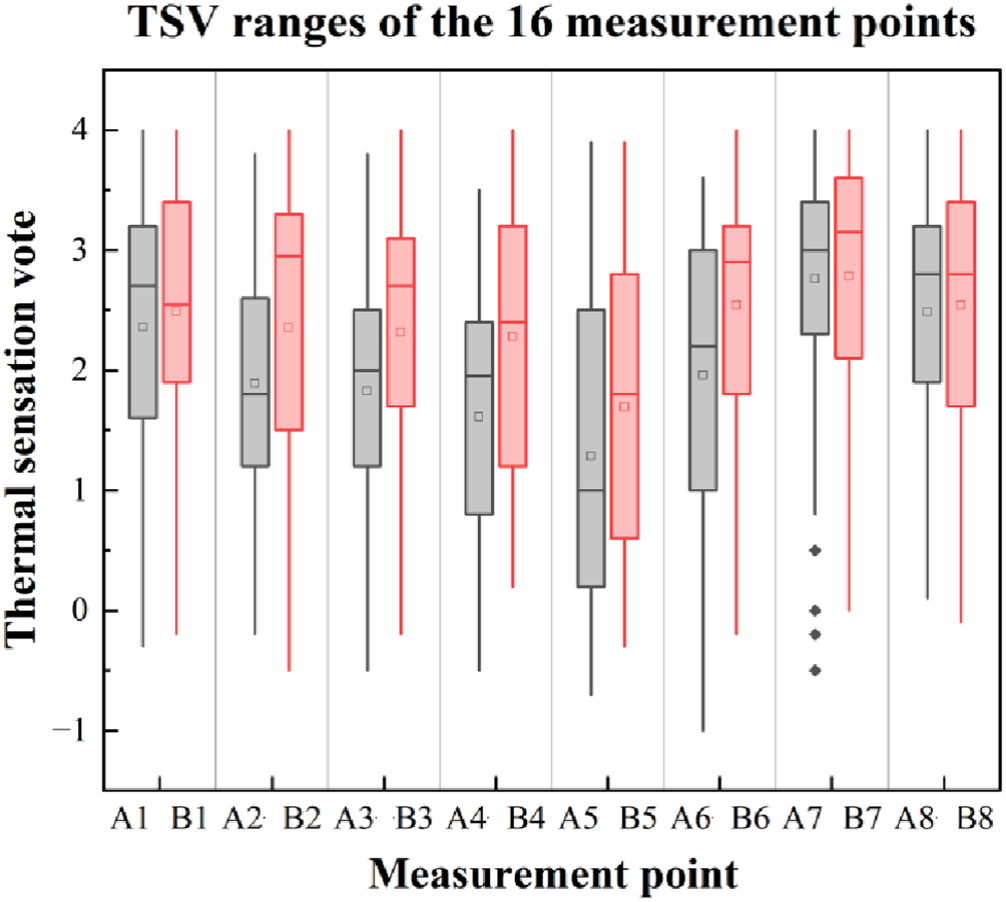
TSV ranges of the 16 measurement points.

Figure 10 depicts the Boxplots of TSV ranges at all points. Points measured on the same day were placed nearby for comparison. In fact, the two nearby points had close scopes, which resulted from the effects of the daily weather conditions. As can be seen from the image, Points A had lower values in total, as far as canopies are concerned. People at points 1, 7, and 8 responded similarly at the two points, with mean TSV of 2.5 (A1 & B1), 3 (A7 & B7), and 2.7 (A8 & B8). Point 2 witnessed a considerable difference between Points A (1.8) and B (2.9) on average. Similarly, they ranged differently, while remarkable scopes emerged at Points 5 and 6, nearly from -1 to 4. There were few regulars about their variation revealed. The subject responses were affected by current meteorology and physical environments commonly. Thus, it is hard to explain the properties of the TSV scopes by the site features.

### General neutral points

#### The neutral temperature

**Figure 11:**
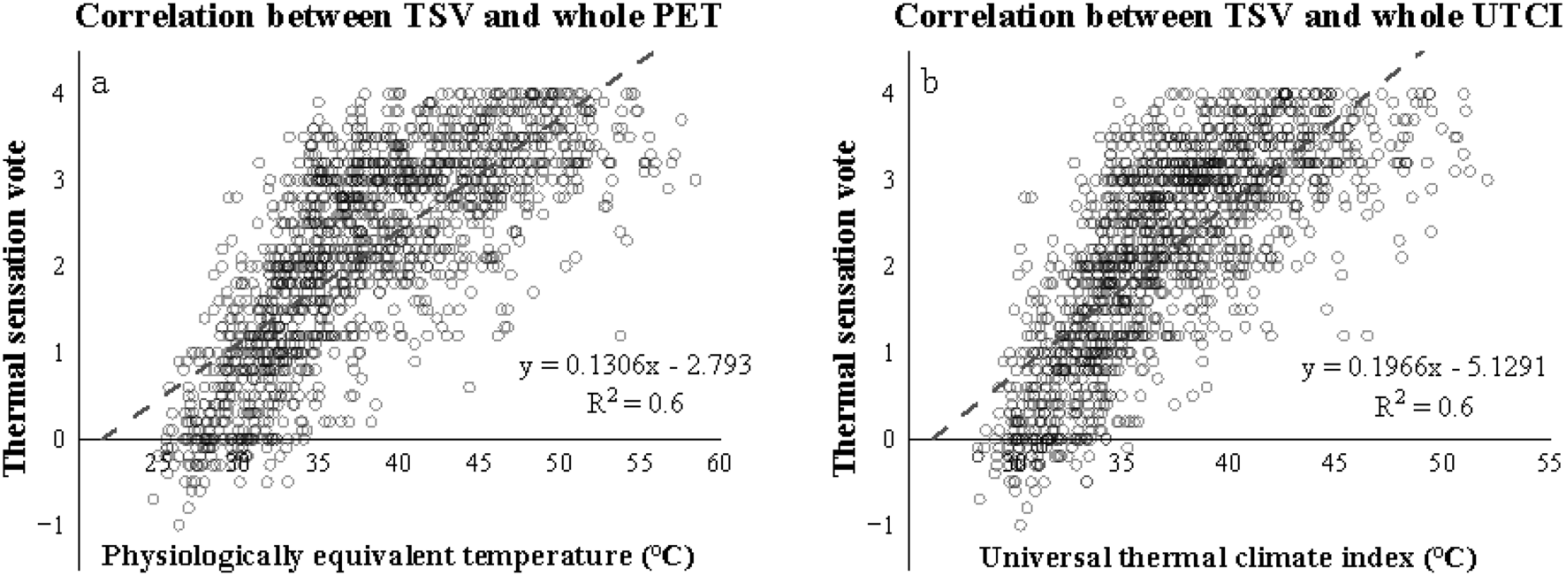
Linear correlations between TSV (**a**, PET; & **b**, UTCI) and thermal indices combining both A & B lines.

The subjective thermal responses were associated with the two OTC indices in separation. As can be seen from the images (Fig. 11), TSV increased as getting warm. The increase of PET by 10 °C caused a TSV rise of around 1.3; in contrast, that of UTCI by the same value increased TSV by 1.9. They were both significantly correlating (R^2^ = 0.6 for PET & 0.6 for UTCI). The NTs obtained from the images were 21.4 °C (PET) and 26.1 °C (UTCI).

**Figure 12:**
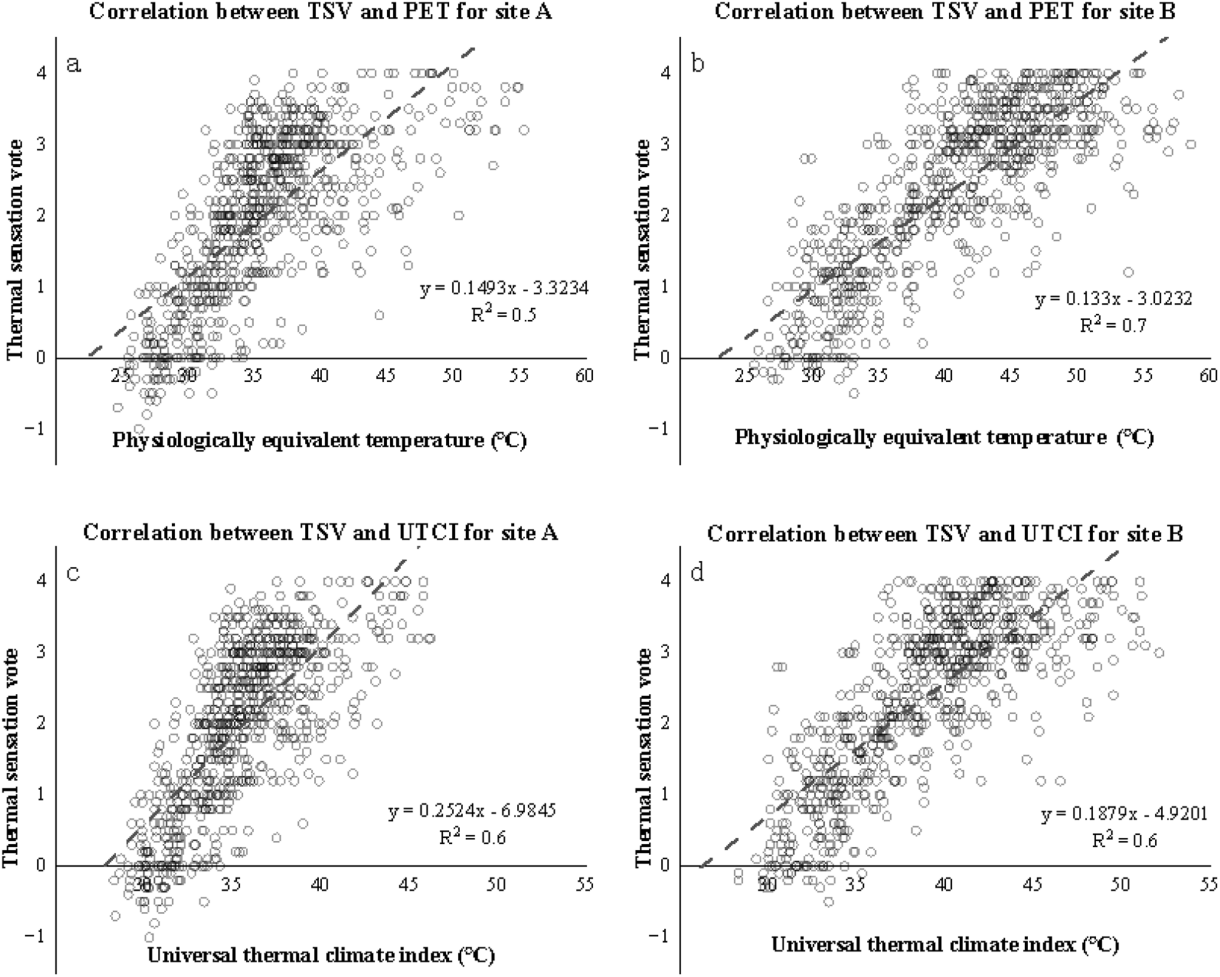
Linear correlation between TSV and thermal indices on the two lines.

As shown in Fig. 12, the TSV of Lines A (shaded) and B (open) are linearly correlated with PET and UTCI. The NT values indicated by PET (NPET) of shaded space and open space were 22.3 °C and 22.7 °C, respectively. In contrast, the NUTCI values (NT indicated by UTCI) were 27.7 °C and 26.2 °C, respectively. For the NPET, the correlation of Line B (R^2^ = 0.7) was greater than that of Line A (R^2^ = 0.5). The sheltered space had a slightly lower neutral temperature (NT) than the open space. For NUTCI, the correlation of Line B (R^2^ = 0.6) was greater than that of Line A (R^2^ = 0.5). The sheltered spaces had higher NTs than the open spaces.

#### Neutral points for wind and relative humidity

**Figure 13:**
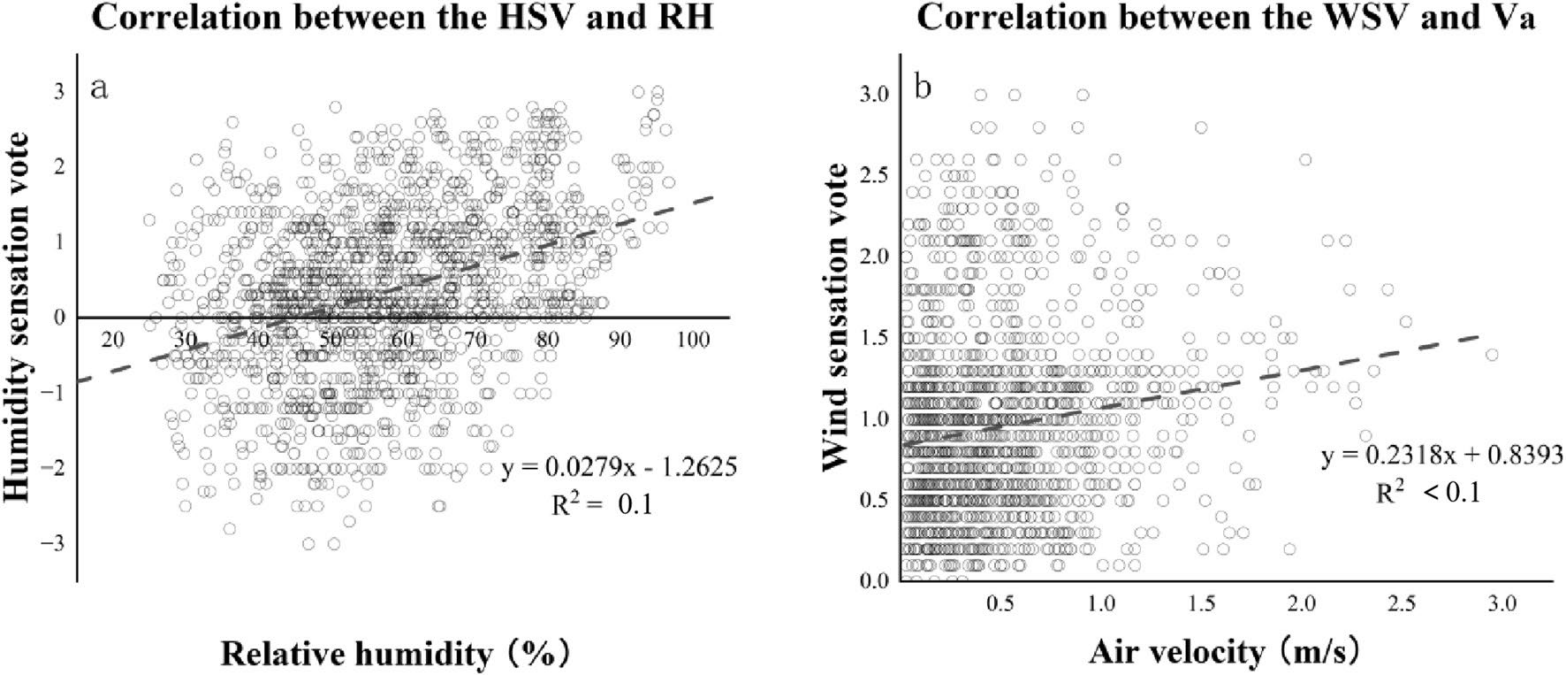
Subjective responses towards various meteorological parameters.

Figure 13 displays the relationship between the RH and humidity sensation vote (HSV) and the relationship between V_a_ and wind sensation vote (WSV). A linear regression of RH against HSV was performed. From our analysis, it was found that the HSV gradually went from very dry (HSV = − 3) to very wet (HSV = 3) as RH increased. At y-axis = 0, the RH was 45.2%, indicating that at this point in time one feels that the RH was neither dry nor wet (HSV = − 3). V_a_ was linearly regressed against WSV. It was found that as the V_a_ increased, the WSV also gradually went from no wind (WSV = 0) to high wind (WSV = 4). However, the correlations were poor in both two models (R^2^ < 0.1 & 0.1).

### The neutral temperature in various contexts

#### The neutral temperature varying for distance from the river

**Figure 14:**
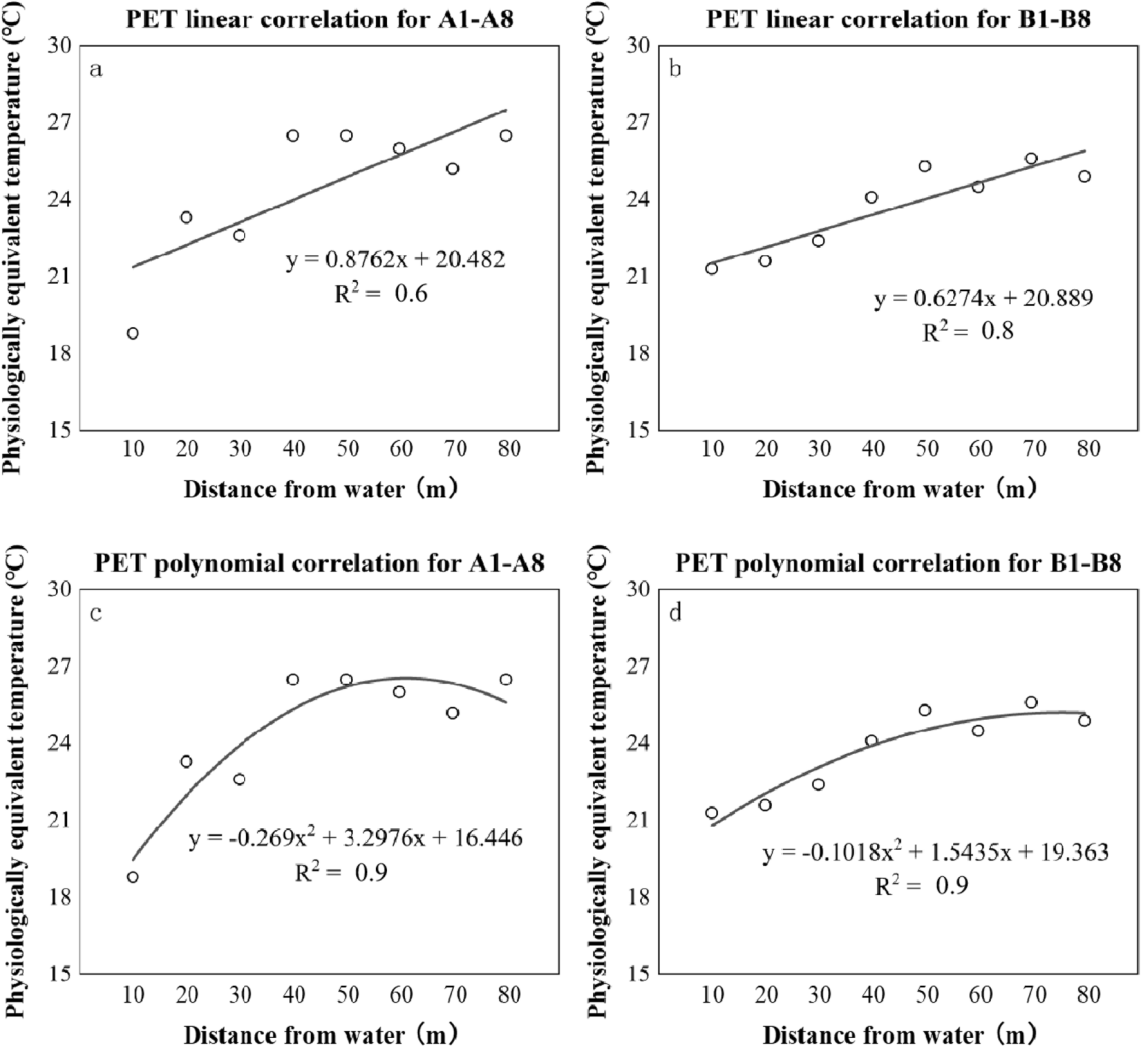
Linear & polynomial correlations between NPETs and DFW (m).

**Figure 15:**
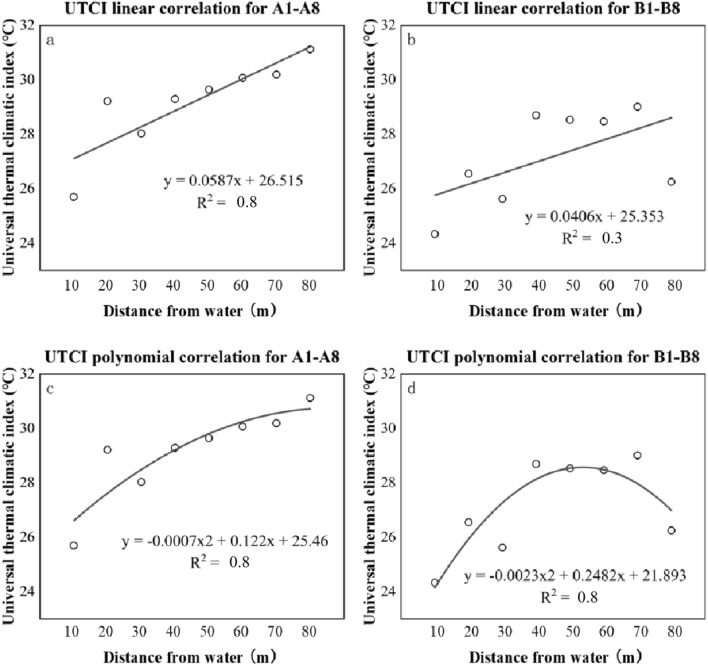
Linear & polynomial correlations between NUTCIs and DFW.

In this work, it was assumed that the water surfaces, as a microenvironmental factor affecting meteorology, would affect subjective thermal perceptions (represented by NTs). The intensities of the influencing varied for distance. Table 6 shows NPET and NUTCI at the 16 points. NTs increased with DFW. The highest value of NPET was found in A4, A5, and A8 (NPET = 26.5 °C), and the lowest value was found in A1 (NPET = 18.8 °C). The highest NUTCI value appeared in A8 (NUTCI = 31.1 °C), and the lowest NUTCI value appeared in B1 (NUTCI = 24.3 °C). Therefore, it can be inferred that people had lower NTs if being closer to the river. Nevertheless, the river would have limited this perceptive effect since its affecting intensity was steady when DFW was above 40 m (Fig. 14). Similar trends were witnessed for NUTCI (Fig. 15).

**Table 6:** NT of all measuring points.

Measuring points	The correlation between TSV and PET	NPET (°C)	DFW (m)	The correlation between TSV and UTCI	NUTCI (°C)
A1	y = 0.1229x − 2.305 (R^2^ = 0.6)	18.8	10	y = 0.216x − 5.5484 (R^2^ = 0.7)	25.7
A2	y = 0.1137x − 2.6517 (R^2^ = 0.6)	23.3	20	y = 0.2099x − 6.1302 (R^2^ = 0.6)	29.2
A3	y = 0.143x − 3.2345 (R^2^ = 0.3)	22.6	30	y = 0.2589x − 7.2557 (R^2^ = 0.3)	28.0
A4	y = 0.2154x − 5.6978 (R^2^ = 0.5)	26.5	40	y = 0.3392x − 9.9355 (R^2^ = 0.5)	29.3
A5	y = 0.3454x − 9.1446 (R^2^ = 0.8)	26.5	50	y = 0.5451x − 16.159 (R^2^ = 0.8)	29.6
A6	y = 0.2449x − 6.3653 (R^2^ = 0.8)	26.0	60	y = 0.4107x − 12.35 (R^2^ = 0.7)	30.1
A7	y = 0.1788x − 4.5784 (R^2^ = 0.7)	25.2	70	y = 0.326x − 9.8431 (R^2^ = 0.7)	30.2
A8	y = 0.2728x − 7.2275 (R^2^ = 0.6)	26.5	80	y = 0.4877x − 15.171 (R^2^ = 0.6)	31.1
B1	y = 0.1302x − 2.7669 (R^2^ = 0.6)	21.3	10	y = 0.1777x − 4.3232 (R^2^ = 0.6)	24.3
B2	y = 0.1165x − 2.5208 (R^2^ = 0.8)	21.6	20	y = 0.1859x − 4.9355 (R^2^= 0.8)	26.6
B3	y = 0.1086x − 2.4304 (R^2^ = 0.6)	22.4	30	y = 0.1568x − 4.0174 (R^2^ = 0.6)	25.6
B4	y = 0.1415x − 3.4115 (R^2^ = 0.7)	24.1	40	y = 0.2509x − 7.1995 (R^2^ = 0.7)	28.7
B5	y = 0.1479x − 3.7409 (R^2^ = 0.7)	25.3	50	y = 0.2124x − 6.0598 (R^2^ = 0.7)	28.5
B6	y = 0.1675x − 4.1028 (R^2^ = 0.8)	24.5	60	y = 0.27x − 7.6857 (R^2^ = 0.8)	28.5
B7	y = 0.1887x − 4.754 (R^2^ = 0.6)	25.6	70	y = 0.2804x − 8.1339 (R^2^ = 0.6)	29.0
B8	y = 0.1534x − 3.7583 (R^2^ = 0.7)	24.9	80	y = 0.1648x − 4.3252 (R^2^ = 0.7)	26.3
NT at line A points	y = 0.1493x − 3.3234 (R^2^ = 0.5)	22.3	NT at line A points	y = 0.2524x − 6.9845 (R^2^ = 0.5)	27.7
NT at line B points	y = 0.133x − 3.0232 (R^2^ = 0.7)	22.7	NT at line B points	y = 0.1879x − 4.9201 (R^2^ = 0.6)	26.2
All of NT	y = 0.1306x − 2.793 (R^2^ = 0.6)	21.4	All of NT	y = 0.1966x − 5.1291 (R^2^ = 0.6)	26.1

#### The neutral temperature varying for relative humidity

**Figure 16:**
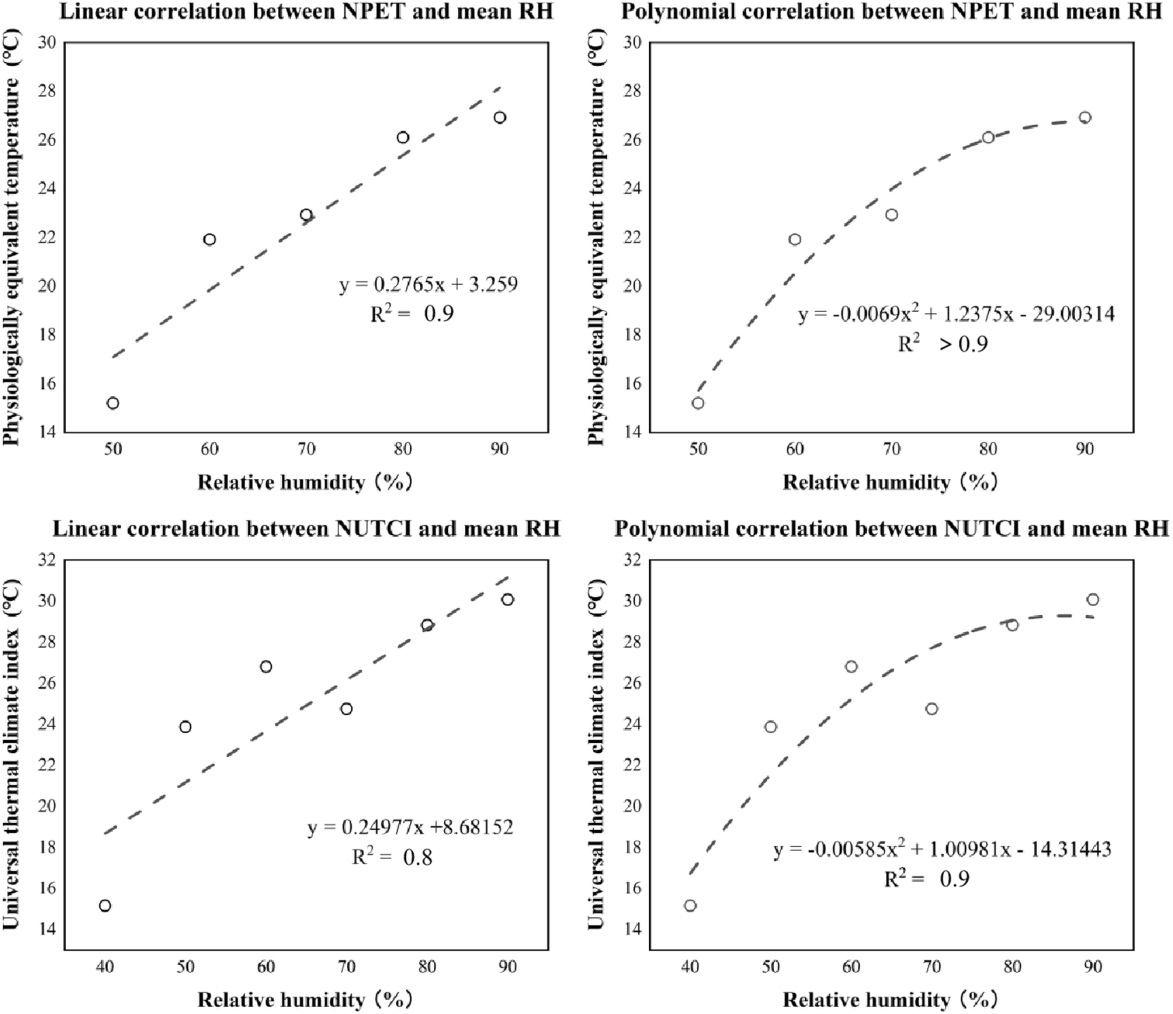
Linear correlation between NTs (indicated by UTCI & PET) at different RH conditions.

According to the above-mentioned analysis, it was found that people responded differently towards DFW. As far as it concerns the cooling properties of water surfaces (air humidity adjustment^53^), the finding might result from the variation of RH. This study assumed that NTs would vary for RH. Therefore, the associations between TSV and PET were analyzed by various RH contexts. The RH values were divided into several groups regarding values in measurements (35–95%, 10% inter value). A variety of NT values towards certain RH ranges were acquired, while some unusual results were obtained (NTs below 0). This might result from special or extreme meteorological conditions. The unusual data were excluded in the further analyses, outputting Fig. 16. The overall trends showed insignificant variation. NTs increased from 15.0 to 27.0 °C as RH raised from 50.0 to 90.0%.

#### The neutral temperature varying for air velocity

**Figure 17:**
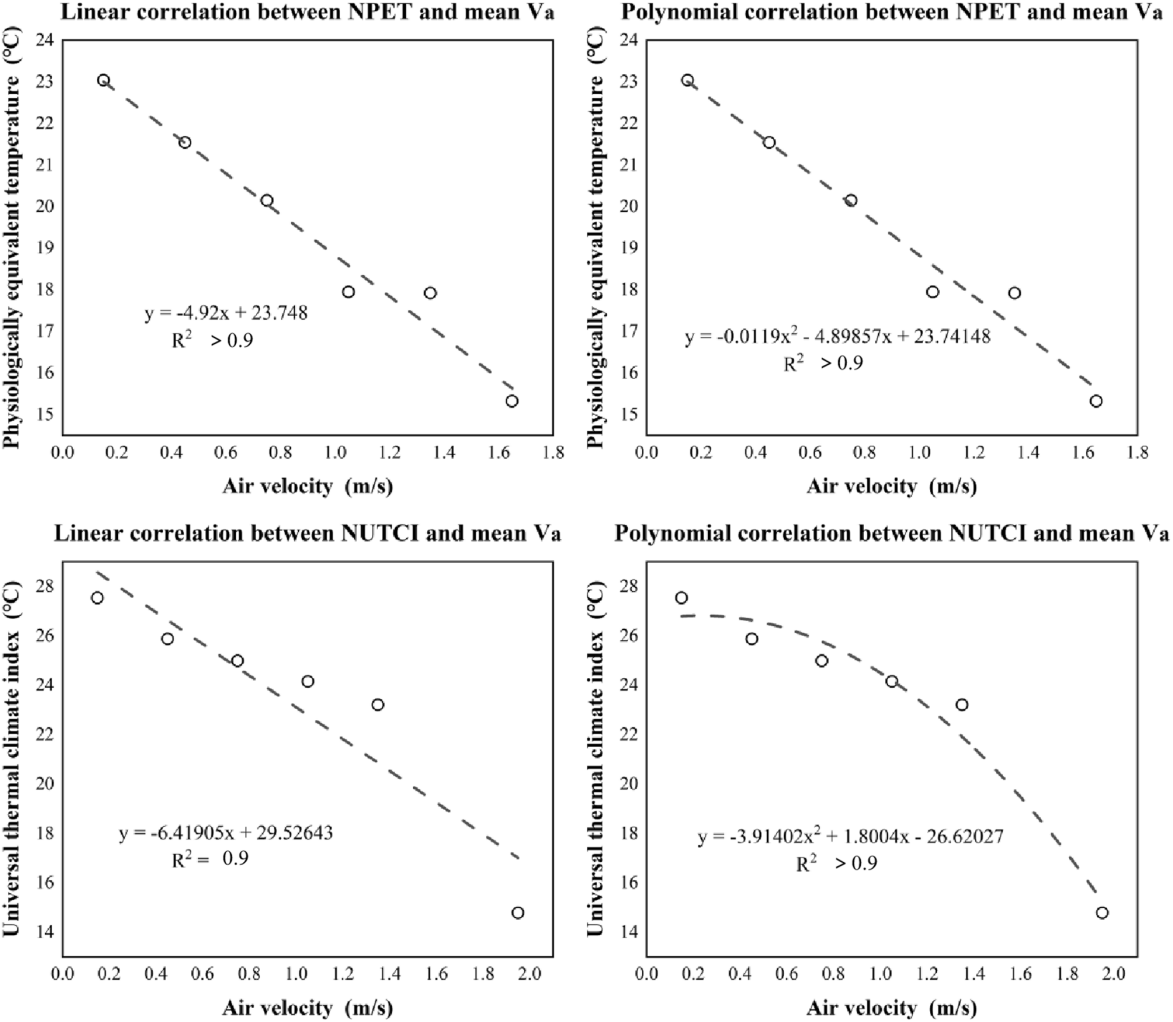
Linear correlation between NTs (indicated by UTCI & PET) at different V_a_ conditions.

People’s thermal perceptions would be affected by other meteorology parameters. NTs were also clustered into various scopes regarding the wind conditions. The measured V_a_ ranged from 0.02 to 2.95 m/s. They were grouped with an interval of 0.3 m/s. With some extreme values excluded (NT values below 0, same as Fig. 17), Fig. 17 was derived. People were increasingly thermal tolerated as getting windier, which could be explained by the lower NT values. The NTs decreased from 23.0 (PET) and 27.0 (UTCI) °C at V_a_ of 0.1 m/s to 15.0 (PET) and 14.0 (UTCI) °C at V_a_ of 1.7 m/s. Significant linear correlations were also found.

## Discussion

The impact of various physical (trees and a water surface) and meteorology factors (RH and V_a_) on subjective thermal perception in a Cfa climate city (as assessed by NTs) was systematically explored here. The NTs (indicated by PET & UTCI) varied for all points and showed a polynomial correlation with contextual parameters. Similar phenomena were witnessed in meteorological context variation. This resulted from various reasons.

People’s thermal responses varied for site openness. As far as PET is concerned, the sites covered by trees had lower NTs. The impact of SVF on OTE/C has been widely examined in the literature. Sites more densely canopied were usually cooler. Song^54^ found that MRT increased by 2.7 °C linearly since the SVF rise by every 0.1 point. Pedestrians might adapt to locations with various SVF thermally. However, NTs in this study were non-linearly correlating with SVF. This implies that the impact of openness on subjective perceptions was complex, which is in line with the reported results of Xiong et al.^27^. and Zhang et al.^55^ and Zhang et al.^23^. They have found, subjective thermal perceptions varied for SVF, yet not always linearly correlating.

People’s thermal perceptions varied also for DFW. The water surface undeniably affects the meteorology conditions for surrounding areas^38^, depending on their distances^25^. More specifically, the sites located too far from the water surfaces would be beyond the affecting ranges of them. Du et al.^56^ found that land surface temperature kept generally steadily as moving farther from the river although it linearly rose at river nearby areas. Consequently, the heat index would polynomially rise with the DFW increase. The polynomial changes of subjective thermal responses (NTs) towards DFW were revealed here, which highlight the effect of water surface cooling and individual thermal adaptation (Fig. 13).

The thermal responses of humans varied for meteorology. This study has found that NTs varied for RH and V_a_. The NTs reduced for V_a_, which was confirmed by Zhang and Lin^57^ and Hou^58^. Generally, windy conditions would cause less heat stress. The wind would be improve the water evaporation^59^, which is cooling effective^60^, reducing heat stress. In other words, people would feel cool under warm if being windy^61^. This phenomenon meets the results of the present study. Furthermore, their NTs varied differently in RH levels. The RH has been proven to be effective on examining thermal comforts^62^. Spaces with higher RH would have denser water molecular weight, which absorbs heat energy in the atmosphere, and lead to a reduced heat stress^63^. In other words, personal heat stress would be reduced by the water molecules during hot seasons^64^. Nevertheless, the underlying origins of this effect are still in controversy. There should be positively or negatively influential^65^.

Overall thermal responses (NTs) were found in this work. They were indicated by PET and UTCI. OTC in Mianyang and some cities with different climates. have been frequently studied in recent years and compared in Table 7. The lowest and highest NTs were found by Cheng et al.^66^ and Xiong et al.^27^, respectively. A relatively low NT value was found in this work. This might result from the water cooling effects and human thermal adaption. According to the literature, the test points mainly focused on the representative area of crowd activity^67^ or the point with the largest flow of people^66^. On this basis, the study expanded the selection range of distance from the water (DFW ≤ 80 m). The changes in the neutral temperature (NT) and distance from water (DFW) were studied, and the range of influence of the water on the heat perception of humans on the shore was determined.

**Table 7:** Comparative study of NT in Mianyang city.

City	Research season	Study area	Summer NPET (°C)	Summer NUTCI (°C)	Reference
Mianyang	Summer, winter	University campus	22.8	–	Huang et al.^67^
Mianyang	Summer, autumn, winter	Mountain parks	17.3	–	Cheng et al.^66^
Mianyang	Summer, winter	Sites around the lake in campus	28.4	–	Xiong et al.^27^
Shanghai	Summer	Hongqiao airport	26.0	27.8	Lian et al.^68^
Tehran	Summer	University campus	20.9	20.7	Haghshenas et al.^69^
New Delhi	Summer	Religion	24.7	22.9	Manavvi and Rajasekar^70^
Mianyang	Summer	Fujiang River	21.4	26.1	This study

Some shortcomings exist despite the study's important findings. Only polynomial correlations between NTs and contextual factors were found. In fact, the involved factor (DFW) was perceptively thermal effective as its original cooling effects^38^. Other influential factors, e.g., SVF and parameters indicating individual trees, were insignificantly explored. Correspondingly, the influential distances would be affected by river properties, such as width and depth, which were not considered. These factors might be perceptively influential as they were broadly proved meteorological impactive (SVF; tree crown dimension; river width). Nevertheless, some unusual phenomena were reported in Sections 3.3.2 and 3.3.3. The NTs were found to be below 0 at some special contexts. This might result from the data errors. There should be limited questionnaire sheets collected in the extreme conditions of either RH or V_a_. This would cause extraordinary analysis results. These issues would be addressed by future studies.

## Conclusion

The study has investigated the impact of various factors on subjective thermal perceptions in riverside spaces. The results indicated that the mean NTs in Mianyang's riverside during the summer of 2023 were 21.4 °C (PET) and 26.1 °C (NUTCI). In sheltered spaces, the NTs were 22.3 °C (PET) and 27.  °C (NUTCI), while in open spaces they were 22.7 °C (PET) and 26.2 °C (UTCI), respectively.

There have been advancements in understanding outdoor thermal comfort from this study, revealing variations in thermal responses under different environmental conditions, which could be a significant step forward in this field, such as Zhang et al.^71^ confirming the correlation between objective thermal conditions and physical environments. This study has used the subjective perception index (NTs) to evaluate the thermal adjusting effects of physical factors (SVF & DFW).

Through combining field measurements with questionnaires, a total of 1669 questionnaire sheets were collected, with 1617 valid responses processed using linear regression to analyse people’s thermal perception differences between various conditions. Key findings included: (a) The mean NPET in Mianyang's riverside during the summer of 2023 was found to be at around 21.4 °C (26.1 °C for NUTCI); (b) When DFW was less than 40.0 m, NTs gradually increased; if DFW exceeded 40.0 m, NTs fluctuated insignificantly; (c) V_a_ was negatively correlated with NTs whereas RH was positively correlated. Perceptive effects of water surfaces and relevant meteorological parameters might be limited. People’s comforts would be adjusted by within certain ranges.

The practical implications of these findings can be applied to urban planning and environmental designs based on contextual considerations such as proximity to the river, wind conditions, and humidity levels.

### Supplementary Information

Supplementary Information.

## Data Availability

The original data generated or analysed during this study are included in this published article as the supplementary (the excel file named as ‘The original data 1214’) file.
